# Hybrid LQR-H_2_ Control of a Kestrel-Based Ornithopter with a Nature-Inspired Flow Control Device for Gust Mitigation

**DOI:** 10.3390/biomimetics11020109

**Published:** 2026-02-03

**Authors:** Saddam Hussain, Ali Hennache, Nouman Abbasi, Dajun Xu

**Affiliations:** 1 Department of Intelligent Systems and Control Engineering, Beihang University, Beijing 100191, China; 2 Engineering Sciences Research Center (ESRC), Deanship of Scientific Research (DSR), Imam Mohammad Ibn Saud Islamic University (IMSIU), Riyadh 11432, Saudi Arabia; ashennache@imamu.edu.sa; 3 Department of Mechanical Engineering, National University of Sciences and Technology, Islamabad 46000, Pakistan; nomi1257@gmail.com; 4 School of Astronautics, Beihang University, Beijing 100191, China; xdj@buaa.edu.cn

**Keywords:** biomimetic aerodynamics, fluid structure interaction, ornithopter flight control, gust alleviation, nature-inspired flow control, flapping flight, bond graph

## Abstract

Unsteady atmospheric disturbances significantly compromise the stability of ornithopters, necessitating advanced turbulence-mitigation strategies. In contrast, natural flyers display remarkable aerodynamic adaptability through dynamic flow-control mechanisms such as covert feathers, enabling stability across unsteady flow regimes. Drawing inspiration from this biological phenomenon, this study presents the modeling and hybrid control of a kestrel-based ornithopter equipped with a Nature-Inspired Flow Control Device (NFCD) that replicates the adaptive feather deployment mechanism observed in kestrels. A reduced-order multibody bond-graph model (BGM) of the full ornithopter is developed, incorporating the main body, propulsion system, rigid wings, and the NFCD subsystem. The model captures key fluid-structure-interaction (FSI) effects between morphing feathers and surrounding airflow. A Linear Quadratic Regulator (LQR) ensures optimal performance under nominal gust conditions (≤3 m/s), while an H_2_ controller activates during high-intensity gusts (≥4 m/s) to enhance disturbance rejection through electromechanical feather actuation. A gain-scheduled transition is employed in the intermediate gust range (3–4 m/s) to ensure a smooth transition between controllers. Simulations indicate up to 70% reduction in gust-induced oscillations and 32% gust-mitigation efficiency, achieved through feather actuation in the NFCD combined with hybrid control, stabilizing the ornithopter in less than 1.4 s under higher gust conditions. The close correspondence between simulated responses and previously reported findings validates the proposed approach. Overall, by merging biomimetic aerodynamics, nature-inspired flow control, and advanced control design, the LQR-H_2_ governed NFCD provides a promising pathway toward gust-tolerant ornithopters capable of resilient and stable flight in unsteady atmospheric environments.

## 1. Introduction

A wide range of unmanned aerial vehicles (UAVs) is gaining popularity, particularly smaller variants such as selfie UAVs, delivery drones, flying robots for power line examination, micro-aerial vehicles (MAVs) for rescue operations, and farming drones [1]. Recently, flapping wing unmanned aerial vehicles (FUAVs), which mimic avian and bird flight, have drawn significant attention from researchers owing to their energy-efficient and agile flight, enhanced resistance to turbulence, and superior maneuverability [2]. However, these UAVs are primarily used at low altitudes, including areas like cities, forests, hills, and coastal regions. Harsh gusts and turbulence in such environments have led to frequent UAV failures and crashes. To enhance mission reliability, it is crucial to develop active gust mitigation systems for these vehicles [3].

Numerous conventional disturbance mitigation approaches for drones have been thoroughly explored. A passive stabilization technique employing a cylindrically shaped aerodynamic device is introduced in [4] to improve flapping wing MAVs (FMAVs) resilience without relying on sensors or feedback. This structure produced uniform corrective moments, allowing recovery from pitch and roll disturbances of ±40° and −75° and vertical displacements nearing 160 mm within one second. When compared to traditional cross-arm dampers, this approach prolonged passive hover duration from 1–2 s to more than 15 s. The method further validated effective disturbance rejection under light gusts (up to 2.6 km/h) through a minimal-weight, nature-inspired solution.

A related study [5] focused on mitigating turbulence effects by accelerating gust detection and improving the reaction speed of the onboard control electronics. The authors employed enhanced signal-processing techniques on MEMS-based gyroscopes to extract disturbance cues more rapidly, leading to noticeably improved stability during gust encounters. While this work demonstrates the value of high-fidelity sensing and rapid disturbance detection, it does not explore how such approaches would translate to flapping-wing UAVs, where unsteady aerodynamics and feather–airflow coupling present fundamentally different control challenges.

One study demonstrates that incorporating synthetic jet actuators in MAVs significantly reduces aerodynamic drag and helps dampen gust-induced oscillations, contributing to improved aerodynamic stability [6]. Another investigation highlights the use of vortex generators to alleviate wing loads during high-speed flight, aiming to prevent wing-tip stall and reduce aerodynamic forces during turbulence and gust events [7]. However, these conventional gust alleviation strategies have primarily been evaluated for fixed-wing aircraft or traditional MAVs, and their applicability to FUAVs remains largely unexamined.

Over the past decade, bio-inspired approaches have rapidly evolved to address challenging engineering problems. Several bio-motivated control strategies have been proposed for stabilizing ornithopters under external disturbances [8,9]. A recent work [10] presents a fly-by-feel control scheme using wing-integrated strain sensors, inspired by insect mechanoreception, to estimate aerodynamic loads without relying on inertial sensors. Reinforcement learning is employed to interpret strain measurements for real-time attitude and airflow estimation. Simulation and flight tests under wind disturbances of 3–7 m/s demonstrate stable and adaptive control of flapping-wing UAVs.

In [11], the authors presented biomimetic flow-sensing elements based on avian feathers for application in fixed-wing MAVs and demonstrated their capability to attenuate gust-induced disturbances. The engineered feather-like structures were installed on both the top and bottom airfoils of the wings, operating concurrently as sensing and actuation components. Experimental results on a prototype fixed-wing MAV confirmed a significant reduction in the effects of incoming gusts.

Alternatively, gust suppression through the use of conventional and learning based controllers is also adopted in flying robots by various researchers [12,13]. A recent study in [14] shows that variable feather overlap enables natural wing morphing through passive elastic compliance of connective tissues, which redistribute feathers during skeletal motion. Micro-scale “directional Velcro” structures on adjacent feathers provide probabilistic locking during extension and unlock automatically during flexion, preserving aerodynamic continuity. Experiments using a feathered biohybrid aerial robot have demonstrated that these passive mechanisms substantially enhance turbulence robustness. Such findings offer valuable design cues for directional fasteners and morphing-wing UAV architectures. Yu et al. [15] proposed an RL-based recovery controller for flapping-wing micro aerial vehicles subjected to extreme attitudes caused by aggressive maneuvers and wind disturbances. A hybrid RL-PD strategy was introduced to ensure post-recovery stability. Simulation results confirmed effective recovery and stabilization under challenging conditions, with experimental validation planned as future work.

Another study [16] introduced a wind velocity assessment scheme together with a dynamic wind model capable of capturing turbulent conditions. A deep reinforcement learning (DRL) method was applied and validated through both simulations and experimental tests, resulting in approximately a 65% reduction in trajectory tracking error and improved UAV stability under wind disturbances up to level 5.

In [17], an LQR-based controller was applied to track the trajectory of an insect-scale flapping drone operating at speeds up to 25 cm/s; however, the analysis was limited to minor disturbances and did not address gust mitigation. In [18], an LQR scheme was proposed for attitude regulation of a flapping drone in gusty conditions, where closed-loop control was identified as essential for maintaining stability and flight-envelope integrity. The approach successfully stabilized the vehicle under wind gusts of up to 3 m/s.

Inspired by birds’ feathers, the study in [19] proposes a bio-inspired feather-based wing design for flapping wing UAVs to dissipate gust effects in turbulent airflows. They presented the model of a wing incorporated with feathers, and simulation results showed that the wing with feather design handles gusts better than the wing without feathers. This work is further extended in [20], which demonstrates that an H-infinity controller can stabilize the unstable wing having feathers in it with robust behavior but inefficient control input usage. Further, in [21], the authors present the H-infinity-LQR composite controller strategy with an observer design and achieve control input efficient robust performance results.

Fisher et al. [22] investigated kestrel-inspired wind-hovering in urban updrafts using an unpowered fixed-wing micro air vehicle (MAV), demonstrating that small MAVs can exploit localized orographic lift to maintain altitude in the presence of turbulence. Their platform was a flying-wing sailplane equipped with onboard sensing and a cascaded PID control architecture designed to hold the vehicle on a precomputed soaring trajectory. The study highlights the biological principle of kestrel wind-hovering but applies it to a non-flapping, non-morphing airframe, where control relies purely on conventional elevon-based attitude regulation rather than on active feather or wing actuation. While this work establishes the feasibility of kestrel-inspired station-keeping for gliding MAVs, it does not address the aerodynamic or control complexities of feather-airflow interaction, multi-degree-of-freedom wing dynamics, or gust-mitigation control for flapping-wing platforms.

Existing control strategies have proven effective primarily for handling mild disturbances and low-speed winds, but they struggle to manage stronger gusts effectively. This is largely because most approaches attempt gust mitigation in isolation, without incorporating active aerodynamic design elements to support control performance. The study in [23] also observed this limitation, noting that single-controller schemes are inadequate for flapping-wing drones operating in dynamic and highly nonlinear environments. They recommend integrating active structural features such as feather-like mechanisms or employing a multi-layered control framework with parallel modules to achieve more robust gust rejection.

**Figure 1:**
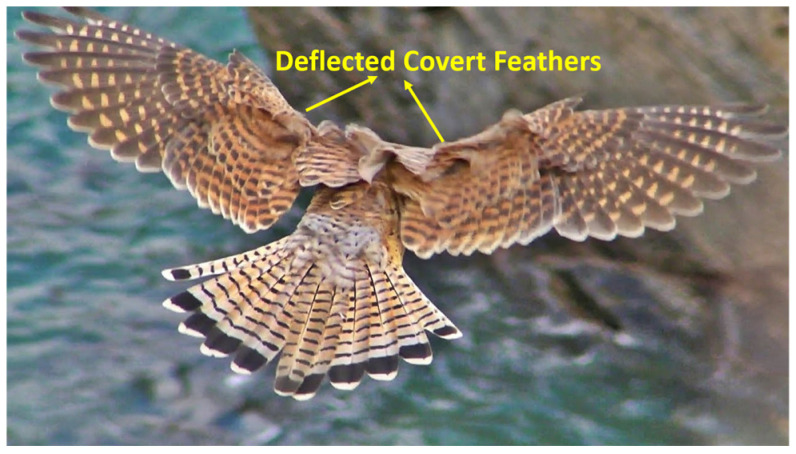
Kestrel’s covert feathers deflected in response to a gust.

This challenge has prompted a shift in focus toward natural flyers, whose evolved strategies may hold the key to effective gust handling. Extensive studies on natural flyers have shown that they often switch to intermittent flight patterns when encountering gusty winds and turbulent atmospheric conditions. During these non-flapping phases, birds typically either glide or loiter in the air. It is in these moments that the covert feathers become active, deploying automatically to mitigate strong winds. This behavior is shown in Figure 1 [24].

The authors in [25,26] show that ornithopters are dynamically unstable, which necessitates an active controller design to achieve stable and robust flight under turbulence and gusts. In addition, most kestrel-inspired UAV studies are limited to fixed-wing gliders, where control is achieved through small-angle PID regulation and no flapping or feather dynamics are involved. In contrast, studies on flapping-wing ornithopters with kestrel-inspired covert feathers remain largely unexplored, especially under gust loads. In this study, we build upon these findings and present a novel design of a complete ornithopter integrated with a Nature-Inspired Flow Control Device (NFCD) on both wings, mimicking the kestrel’s covert feathers along with a hybrid LQR-H_2_ controller. This integrated design and hybrid control approach for ornithopters has not been previously explored in the literature and successfully achieves stability and robustness in ornithopter flight under gusty airflows. The key contributions of this work include the following:

• Development of a longitudinal bond graph model of a complete ornithopter with NFCD-equipped wings.
• Implementation of a hybrid LQR-H_2_ control strategy with two primary modes: an LQR controller for stability under nominal flight conditions (gust speed ≤ 3 m/s), and an H_2_ controller coupled with NFCD actuation to maintain performance under high-intensity gusts (gust speed ≥ 4 m/s).
• Design of a gain-scheduled switching mechanism for the intermediate gust range (3 m/s < gust speed < 4 m/s) to enable smooth transitions between control regimes.
• Validation of the hybrid LQR-H_2_ controller integrated with the covert-feather-based NFCD through comparison with existing literature, confirming the reliability and effectiveness of the proposed approach.

While LQR and H_2_ controllers have been individually explored in UAVs and flapping-wing systems, the literature lacks any integrated framework that combines a hybrid LQR-H_2_ strategy with an active covert-feather-based NFCD on a kestrel-inspired ornithopter. Previous studies have either applied LQR for mild gust stabilization, explored H_2_ control for rigid-wing configurations, or proposed passive feather-inspired mechanisms. However, no prior work has developed a reduced-order multibody bond graph model of an ornithopter equipped with electromechanical covert feathers and synthesized a gain-scheduled hybrid controller that adapts to gust intensities. This integrated, bio-inspired, and control-oriented architecture constitutes the primary novelty of the present study and distinguishes it from existing approaches in the literature.

The remainder of the paper is organized as follows: Section 2 describes the design of an ornithopter equipped with NFCD and details the formulation of the bond graph model. Section 3 presents the control-oriented model, while the stability analysis is covered in Section 4. Section 5 outlines the design of the hybrid controller. Section 6 discusses the simulation results, and Section 7 concludes the paper.

## 2. Ornithopter Design and Mathematical Modeling

### 2.1. NFCD-Based Ornithopter Design

**Figure 2:**
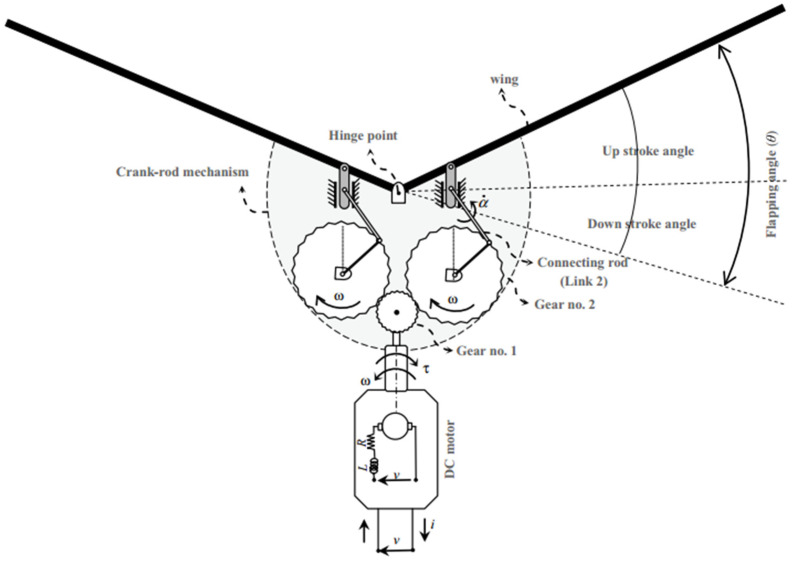
Sketch of the present flapping mechanism based on FESTO Bird.

The Festo Flapping Bird [27] is selected as the baseline ornithopter for this study. The sketch of the present flapping mechanism based on the FESTO Bird is given in Figure 2. The ornithopter platform comprises a fuselage length of approximately 1.07 m and a total wingspan of 2.2 m, with an average wing chord of 0.28 m. The overall mass of the vehicle is about 650 g. It is powered by a 7.4 V battery rated at 450 mA, and the estimated onboard power consumption is around 23 W. The airframe is constructed using a lightweight carbon-fiber composite to ensure structural rigidity with minimal weight.

**Figure 3:**
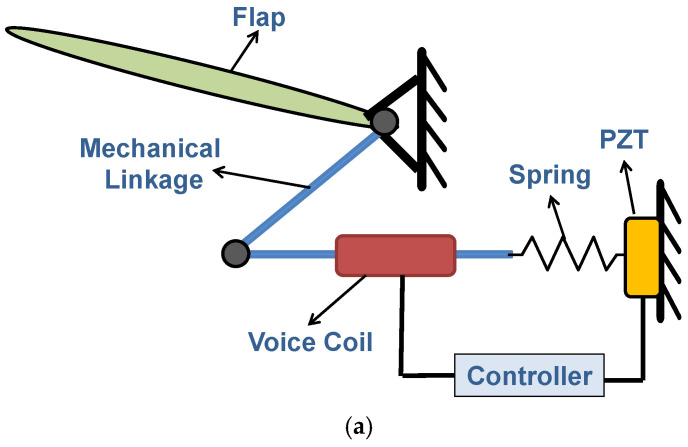

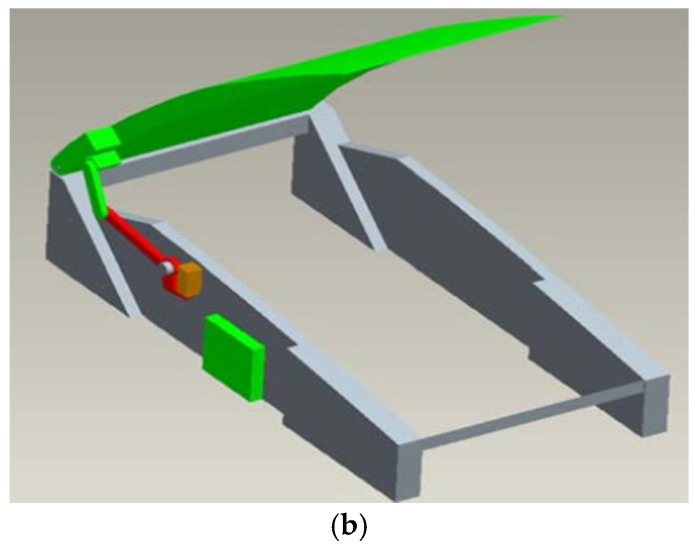
Electromechanical covert feather. (**a**) Schematic diagram. (**b**) CAD diagram.

The proposed ornithopter is composed of subsystems, namely the main body, motors, the flapping mechanism, rigid wings, and the Nature-Inspired Flow Control Device (NFCD). The NFCD incorporates a total of 8 electromechanical (EM) covert feathers and is installed in each ornithopter wing. Each EM covert feather includes a flap, a mechanical linkage, a voice coil actuator, a bio-inspired controller, a spring, and a piezoelectric transducer (PZT). The configuration and the CAD diagram of the EM feathers are illustrated in Figure 3. The flap rotates under a vertical gust and transfers motion through the spring and mechanical link to the PZT. The PZT generates a voltage proportional to the gust and sends it to the bio-mimetic controller, which computes the control input. This current drives the voice coil actuator, whose shaft applies force back on the flap via the linkage to create the required deflection. Thus, the gust transpires with minimal interaction on the wing.

The proposed NFCD enables localized and adaptive regulation of aerodynamic loads along the ornithopter’s wings. Each EM feather functions as an independent flow-control unit, responding instantly to gusts through distributed actuation. This localized adaptation reduces spanwise load fluctuations, delays flow separation, and enhances lift stability. By replicating the hierarchical control of kestrel’s covert feathers, the NFCD achieves lightweight, energy-efficient gust alleviation consistent with the principles of biomimetic aerodynamic design.

Beyond the functional description above, the electromechanical covert-feather unit exhibits a well-defined force-displacement pathway governed by the hinge stiffness, PZT stack compliance, and voice-coil electromechanical coupling. A vertical gust induces a flap rotation that is transmitted through the mechanical linkage, generating a corresponding deformation of the PZT stack. The PZT equivalent stiffness and the hinge stiffness determine the instantaneous mechanical impedance of this conversion. The resulting voltage drives the voice-coil actuator, whose force constant and viscous damping convert the electrical input into a restoring force that regulates the flap deflection. The parameter values of the NFCD-based ornithopter are appended in Table 1.

The latency of this actuation loop is dominated by the mechanical mass-spring-damper dynamics of the flap-hinge-PZT assembly and is on the order of a few milliseconds, consistent with previously reported bench-level measurements of similar piezoelectric/voice-coil systems. The bandwidth is similarly defined by the inertia I_1_, stiffness C, and damping R, yielding a rapid transient response suitable for real-time gust regulation. The NFCD parameters used in the present model, including stiffness, damping, and force-constant values, are directly sourced from earlier experimental characterizations, ensuring that the simulated NFCD behavior remains physically grounded while maintaining a reduced-order structure suitable for control synthesis. The combined PZT–voice coil actuation loop exhibits a mechanical latency on the order of 2–5 ms and an effective bandwidth in the 150–300 Hz range, consistent with previously reported measurements for lightweight feather-actuation systems and sufficient for real-time gust-response regulation.

The added mass of the NFCD module is minimal because each electromechanical feather element weighs only a few grams, and the overall mass increase relative to the baseline ornithopter is negligible. At this scale, the NFCD does not meaningfully alter the wing’s inertial properties or the flapping energetics. The associated electrical power consumption of the PZT–voice coil unit is also low due to its small displacement range and high mechanical stiffness. For this reason, the mass and power penalties are not explicitly modeled in the reduced-order control formulation in this research.

### 2.2. Bond Graph Modeling

Bond graphs offer a powerful, domain-independent graphical approach for modeling the dynamic behavior and energy exchange in physical systems. In contrast to conventional methods that are typically confined to a single domain, bond graphs provide a unified framework capable of representing systems across mechanical, electrical, hydraulic, thermal, and other energy domains. This modeling technique is particularly well-suited for complex, multi-domain systems like ornithopters, where coordinated behavior among mechanical structures, actuators, and aerodynamic forces must be accurately captured. Moreover, bond graphs naturally lend themselves to generating state-space representations, facilitating seamless integration with control design and simulation processes [28].

Given the multi-domain nature of the proposed ornithopter, bond graph modelling is employed in this study to derive its mathematical representation. 20-SIM 4.0 simulation software is used for the development of BGM. The ornithopter is composed of subsystems, namely the main body, propulsion system (motors and flapping mechanism), rigid wings and NFCD.

#### 2.2.1. Rigid Body

**Figure 4:**
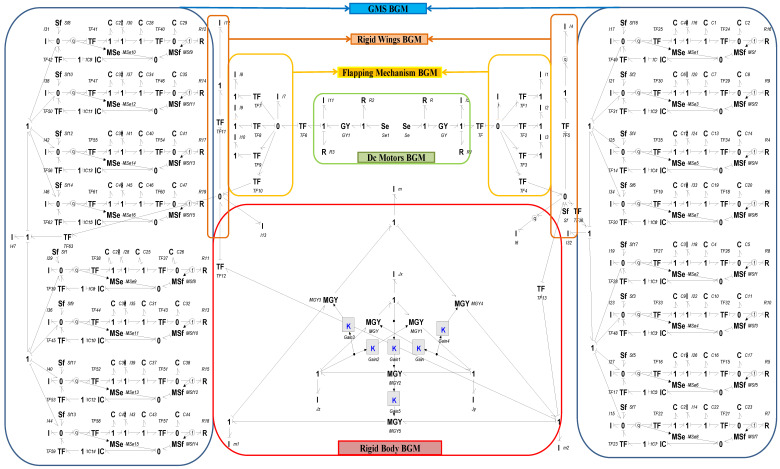
BGM of a complete ornithopter installed with NFCD.

The ornithopter’s body is presented as a 6-DOF rigid structure that can complete both translational and rotational motions. The investigation of a rigid body’s motion generates equations appended below derived from Euler’s equations [28]. The state matrix contains generalized momentum at every inertia element.

(1) $$\dot{p_{x}}=F_{x}+{m\omega }_{z}\frac{P_{y}}{m}-{m\omega }_{y}\frac{P_{z}}{m}$$

(2) $$\dot{p_{y}}=F_{y}+{m\omega }_{x}\frac{P_{z}}{m}-{m\omega }_{z}\frac{P_{x}}{m}$$

(3) $$\dot{p_{z}}=F_{z}+{m\omega }_{y}\frac{P_{x}}{m}-{m\omega }_{x}\frac{P_{y}}{m}$$

(4) $$\dot{{pȷ}_{x}}=\tau_{x}+J_{y}\omega_{y}\frac{{PJ}_{z}}{J_{z}}-{J_{z}\omega }_{z}\frac{{PJ}_{y}}{J_{y}}$$

(5) $$\dot{{pȷ}_{y}}=\tau_{y}+J_{z}\omega_{z}\frac{{PJ}_{x}}{J_{x}}-{J_{x}\omega }_{x}\frac{{PJ}_{z}}{J_{z}}$$

(6) $$\dot{{pȷ}_{z}}=\tau_{z}+J_{x}\omega_{x}\frac{{PJ}_{y}}{J_{y}}-{J_{y}\omega }_{y}\frac{{PJ}_{x}}{J_{x}}$$

where F = ($F_{x}\;,\;F_{y}\;,\;F_{z}$) are external forces, τ = ($\tau_{x},\;\tau_{y},\;\tau_{z}$) are external torques, p = (p_x_, p_y_, p) is the linear momentum, and p_j_ = (p_jx_, p_jy_, p_jz_) is the angular momentum. The rigid body dynamics expressed in Equations (1)–(6) can further be cast into the generalized multibody dynamics framework widely used in the literature [29,30,31] to model the body of ornithopters:

(7) $$M\left(q\right)\dot{\;v}\;+\;C\left(q,\dot{q}\right)v\;=\;W\;$$

where ${v\;=[\dot{x},\dot{y},\dot{z},\omega_{x},\omega_{y},\omega_{z}]}^{T}$ is the twist vector, M(q) is the inertia matrix of the rigid body, $C\left(q,\dot{q}\right)$ incorporates the Coriolis and gyroscopic couplings between linear and angular motion and $W\;=(F_{x},F_{y},F_{z},\tau_{x},\tau_{y},\tau_{z}$) is the external wrench. Coriolis and gyroscopic couplings are realized by power-conserving interconnections (GY/MGY/TF) between translational and rotational ports in the BGM, as shown in Figure 4.

#### 2.2.2. Propulsion System

The propulsion system comprises batteries, motors, and a slider-crank mechanism. The two DC motors are driven by batteries and are comprised of an armature having resistance and inductance, and an electromechanical coupling [29]. The ornithopter’s flapping movement is achieved by a slider-crank mechanism actuated by DC motors. The reciprocating motion is received and transmitted through a crankshaft, which helps achieve the transformation of rotational motion into a reciprocating motion and also vice versa [28].

#### 2.2.3. Rigid Wings

Wings’ dynamics are demonstrated as a rigid beam in transverse motion having a pivoted end. The wing’s perpendicular displacement at the end point is obtained by the following [28]:

(8) $$\begin{array}{c} y\;=\;l\sin\theta \end{array}$$

where *l* is wingspan, *y* is displacement, and *θ* is flapping angle. The effort-flow relationship can be given as follows [29]:

(9) $$\begin{array}{c} V_{y}\;=\;(lcos\;\theta )\omega \end{array}$$

(10) $$\begin{array}{c} \left(x_{1}\cos\theta \right)\;F=\tau \end{array}$$

where *τ* is torque, *F* is force, and *V_y_* is vertical velocity [29]. If *m* is the wing’s mass, then the inertial force derived from Newton’s second law will be given as follows:

(11) $$F\;=\;m\;\ddot{y}\;=\;mlcos\;\theta \ddot{\theta }\;-\;mlsin\theta \;{\left(\dot{\theta }\right)}^{2}$$

The wing dynamics expressed in (8)–(11) can further be cast into the generalized multibody wing dynamics framework as follows:

(12) $$M\left(q\right)\;\ddot{q}\;+\;C\left(q,\dot{q}\right)\dot{q}\;=\;F\;$$

where the generalized coordinate is q=θ, the effective inertia term is $M\left(q\right)\;=\;mlcos\;\theta$, the Coriolis/centrifugal contribution is $C\left(q,\dot{q}\right)\;=\;-mlsin\theta \;$ and F is the generalized input force from motor/aerodynamic loads, mapped via transformer elements in bond graph. Wing lift/drag are included as modulated effort sources (MSe) with TF mapping to pitching moment, while the R-elements provide aerodynamic damping.

#### 2.2.4. NFCD Model

The wings of the ornithopter are equipped with kestrel-inspired feather-based NFCD. A model of a single EM covert feather is developed using the component diagram of the EM feather given in Figure 3, and this is further utilized to form the BGM of an NFCD comprising 8 EM feathers. The mathematical equations for a single EM feather, derived from the BGM depicted in Figure 4, are presented in Equations (13)–(20).

(13) $$\dot{p_{1}}=ic\;\cdot p_{3}+ic\cdot q_{3}$$

(14) $$\dot{q_{1}}=\frac{1}{I_{1}}\;\cdot p_{2}$$

(15) $$\dot{p_{2}}=\frac{ic}{l}p_{3}+\frac{ic}{l}q_{3}-\frac{1}{C}q_{1}-\frac{1}{C_{1}}q_{2}-\frac{m}{C_{2}}q_{4}\;$$

(16) $$\dot{q_{2}}=\frac{1}{I_{1}}\;\cdot p_{2}$$

(17) $$\dot{p_{3}}=q_{5}$$

(18) $$\dot{q_{3}}=S_{f}-\frac{1}{l\;\cdot \;I_{1}}p_{2}-\frac{1}{I}p_{1}$$

(19) $$\dot{q_{4}}=\frac{m}{I_{1}}p_{2}-\frac{R}{C_{2}}q_{4}$$

(20) $$\dot{q_{5}}=\frac{1}{l\;\cdot \;I_{1}}p_{2}$$

The state variables in Equations (13)–(20) consist of *p*_1_, *p*_2_, and *p*_3_, which represent the generalized momentum associated with the inertial elements, and *q*_1_, *q*_2_, *q*_3_, and *q*_4_, which denote the generalized displacements corresponding to the compliance elements. *I* is mass of the feather flap, *S_f_* is gust velocity on the flap, *IC* is the voice coil actuator compliance and stiffness, *R* is the resistance between the amplifier and the PZT, *I_1_* is the mass of the stack, *C_1_* is the PZT spring stiffness, *C_2_* is the PZT equivalent capacitance, *TF_1_* is the electromechanical coupling ratio, *C* is the stiffness of the spring, and *TF* is the transformer ratio of the mechanical linkage. Feathers aero-loads (MSe) act on compliant hinges (*C* with *R*), closing the fluid–structure interaction (FSI) loop while the gust perturbs the aerodynamic port as an exogenous Se element. The actuator subsystem is represented through its dominant linear stiffness, damping, and coupling parameters, which is appropriate because the feather deflections remain within the small-angle regime and the PZT–voice coil operates over a narrow displacement range. Under these conditions, the electromechanical behavior is well approximated by a linear model.

#### 2.2.5. Multibody Model of a Complete Ornithopter

The model of a complete ornithopter is established by connecting the BGM of subsystems via appropriate junctions and is depicted in Figure 4. For a detailed component-level development of a complete ornithopter model, further reading of the author’s previous work is suggested [25,26]. The state vector $\overset{⃑}{x}\left(t\right)$ comprises the momenta of the I-elements and the generalized displacements of the C-elements. Linearization about the hover trim condition yields a 132-state model that captures the complete 6-DOF rigid-body dynamics of the ornithopter installed with NFCD in both wings. Since the focus of this study is on flight control synthesis, the analysis is restricted to the longitudinal plane. In this way, only the forward velocity, vertical velocity, pitch angle, and pitch rate are retained, while the lateral directional states are excluded. The resulting subsystem provides a direct pathway to the conventional four-state longitudinal model, which is presented in the next section.

#### 2.2.6. Aerodynamic Modeling

In this research, the aerodynamic formulation is intentionally kept reduced-order and control-oriented to enable tractable hybrid LQR-H_2_ controller synthesis. Although flapping-wing aerodynamics inherently involve complex unsteady phenomena such as leading-edge vortex (LEV) formation, rotational lift, added-mass forces, wake capture, and nonlinear vortex shedding, including such effects requires high-fidelity CFD or aeroelastic FSI solvers that significantly increase state dimensionality. These full-order models are not suitable for real-time control design, especially when integrated with the multibody NFCD dynamics.

Consistent with recent reduced-order flapping-wing modeling frameworks [30,31,32], the present study adopts a quasi-steady aerodynamic approximation embedded within the bond-graph structure. Vertical gust excitation is modelled as an incremental velocity input that generates a quasi-steady lift force in the vertical (z) direction, implemented through a source of flow (Sf) term. In the present study, this gust is represented as a step change in the vertical component of the oncoming flow, with constant magnitude during each simulation run (<15 m/s as analyzed in Section 6). Stochastic gust models such as the Dryden or Kaimal spectra are not considered here and are reserved for future work focusing on broadband turbulence. This aerodynamic load is passed to the elastic wing via a modulated effort source (MSe) and transformed into rigid-body vertical motion using the MTF (modulated transformer) element, as illustrated in Figure 4.

Aerodynamic loads acting on the NFCD feathers scale proportionally with the imposed gust magnitude because the modulated effort sources (MSe) are driven by the gust-induced velocity increment at each feather hinge. This linear dependence is consistent with quasi-steady formulations and ensures that higher gust magnitudes generate correspondingly larger feather loads within the physical limits defined by hinge stiffness and damping.

Higher-order unsteady contributions; LEV dynamics, added-mass terms, wake–wing interactions, and viscous effects are acknowledged omissions. Their exclusion allows the focus to remain on evaluating the hybrid LQR-H_2_ scheme and the NFCD’s gust-mitigation behavior. Future work will incorporate CFD-derived coefficients, surrogate unsteady aerodynamic models, flexible-wing aeroelastic coupling, and multi-axis gust fields for a more comprehensive aerodynamic representation.

## 3. Control-Oriented Model

For the purpose of controller design, the reduced four-state longitudinal model of the NFCD-equipped ornithopter is employed. This representation, obtained at the hover trim condition, captures the dominant forward and vertical velocity dynamics together with the pitch motion, making it well-suited for stability analysis and control synthesis. The model is formulated in a linear time-invariant (LTI) state-space form and serves as the basis for subsequent H_2_ controller design. The state vector x, control vector u and the output vector y are as follows: x =  [ u w θ q]*^T^*, u = [$\;\varphi_{o}\;\alpha_{m}\;\alpha_{o}]$*^T^*, y = [ u w θ]*^T^*. The details of these vectors are given in Table 2. Here, the flapping stroke offset $\varphi_{o}$ biases thrust and primarily affects the forward dynamics, the pitch angle magnitude $\alpha_{m}$ governs the lift and hence the vertical dynamics, and the pitch angle offset $\alpha_{o}$ influences the pitching moment of the NFCD-equipped ornithopter. The resulting LTI system can be represented in state space form as Equation (21). The numerical entries in Equation (21) are obtained directly from the physical parameters listed in Table 1, which were identified and experimentally characterized in the authors’ earlier ornithopter modeling studies [25,26].

(21) $$\begin{array}{c} \dot{\overset{⃑}{x}}\left(t\right)=A\cdot \overset{⃑}{x}\left(t\right)+B\cdot \overset{⃑}{u}\left(t\right)\;\\ \overset{⃑}{y}\left(t\right)=C\cdot \overset{⃑}{x}\left(t\right)+D\cdot \overset{⃑}{u}\left(t\right) \end{array}$$

$$A=\;\left[\begin{array}{cccc} -5.989 & 0 & 0 & -0.069\\ 0 & -1.98 & -8.91 & 0.004\\ 0 & 0 & 0 & 1.30\\ 0 & 149.34 & 0 & -0.298 \end{array}\right]$$

$$B=\left[\begin{array}{ccc} 0 & 17.0 & 0\\ 0 & 0 & -12.7\\ 0 & 0 & 0\\ 1483 & 0 & 597 \end{array}\right]$$

$$C=\left[\begin{array}{cccc} 1 & 0 & 0 & 0\\ 0 & 1 & 0 & 0\\ 0 & 0 & 1 & 0 \end{array}\right]\;\;\;D=\mathrm{zeros}\;(3,3)$$

### Modeling Assumptions and Realism Considerations

The control-oriented longitudinal model employed in this study is derived by linearizing the complete bond-graph formulation about the hover trim condition. This approach is standard practice in ornithopter control design and enables hybrid LQR–H_2_ synthesis without the computational burden associated with full nonlinear or periodic flapping-wing dynamics. To preserve analytical tractability, several practical effects are omitted, including actuator saturation limits, sensor noise, electromechanical time delays, and broadband stochastic gust fields. These factors, while relevant for hardware implementation, are not essential for evaluating the baseline gust-attenuation capability of the hybrid controller.

The gust disturbance is therefore introduced as a deterministic step input to isolate the closed-loop response of the LQR, H_2_, and switching regimes. Similarly, actuator dynamics are represented through their dominant stiffness, damping, and coupling characteristics, as detailed in Table 1, rather than through full bandwidth-limited servo models. These assumptions do not affect the qualitative behavior of the hybrid controller but significantly simplify the analysis and model order.

The limitations of this simplification are acknowledged, particularly for large-amplitude periodic flapping, nonlinear aerodynamic couplings, and actuator bandwidth constraints. Future extensions will incorporate measured sensor-noise profiles, actuator saturation limits, communication delays, and stochastic gust models (e.g., the Dryden or Kaimal spectra), together with nonlinear simulation of the full bond-graph model for hardware-in-the-loop validation.

## 4. System Stability Study

**Figure 5:**
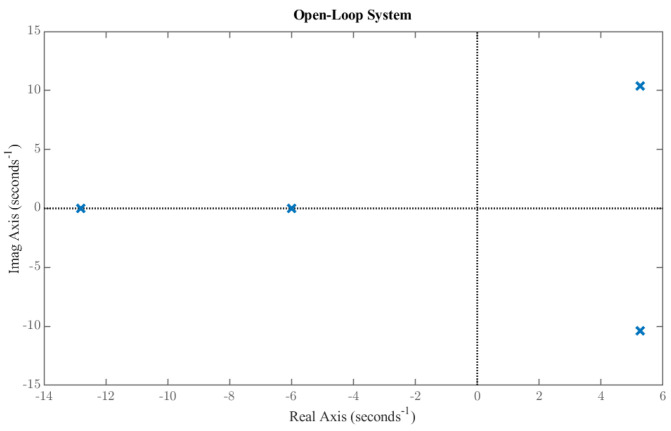
Open−loop pole−zero plot.

**Figure 6:**
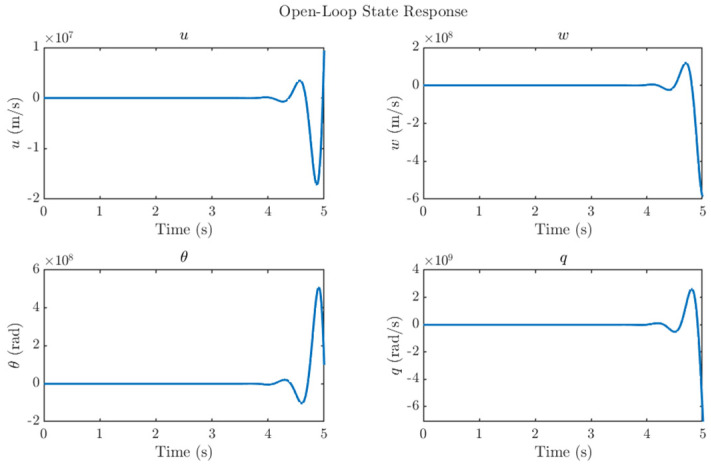
Open−loop states a response to a gust.

Stability analysis of the reduced-order model is presented in this section. The eigenvalues of the open-loop system are located at: −5.98, −12.81, 5.26 ± 10.35j. The presence of a conjugate pair of poles in the right-half-plane, as shown in Figure 5, indicates that the system is inherently unstable. Figure 6 illustrates the unstable state response of the open-loop system, which shows a diverging state response and instability in the system. This instability underscores the need for a hybrid control strategy that combines H_2_ and LQR techniques to ensure attitude stability under both nominal weather conditions and gust disturbances. The system is verified to be fully controllable, with a controllability rank of 4.

## 5. LQR-H_2_ Hybrid Controller Design

**Figure 7:**
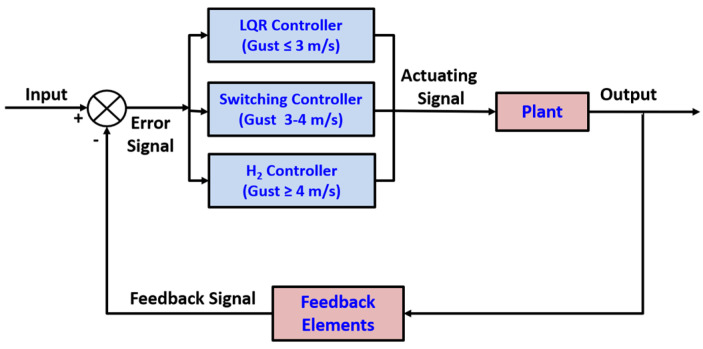
LQR−H_2_ hybrid controller architecture.

In this section, a hybrid control scheme is developed by combining LQR control with H_2_ control, integrated through a gain-scheduling mechanism to enable smooth transitions between the two controllers. The closed-loop architecture of the proposed hybrid controller is depicted in Figure 7. The LQR controller is employed under nominal gust conditions (gust speed ≤ 3 m/s) to ensure optimal performance with minimal control effort. For higher gust intensities (gust speed ≥ 4 m/s), the H_2_ controller is engaged to provide robust disturbance rejection via electromechanical feather actuation inside NFCD. In the intermediate gust range (3 m/s < gust speed < 4 m/s), a switching strategy is employed to transition seamlessly between the two control regimes. A single H_2_ controller is found to be unnecessarily conservative under mild-gust conditions, whereas LQR achieves faster settling and lower control effort in this regime. Likewise, in higher gusts, the LQR controller is found to be less robust and therefore, the hybrid structure exploits LQR efficiency at low gust levels and H_2_ robustness at higher disturbance intensities.

The switching threshold between the LQR and H_2_ regimes is selected based on the transition region where the flapping-wing ornithopter begins to exhibit significant gust-induced deviations in vertical force and displacement. Preliminary simulations indicated that gusts below approximately 3 m/s produce small, rapidly attenuated perturbations adequately handled by LQR alone [18], whereas gusts above 4 m/s generate larger deviations that benefit from the enhanced disturbance rejection of H_2_ control. The chosen 3–4 m/s boundary, therefore, reflects the natural separation between mild and moderate gust response in the reduced-order model rather than an arbitrary selection.

To avoid chattering at the switching boundary, the hybrid controller uses a hysteresis-based switching rule. The controller switches from LQR to H_2_ only when the gust estimate exceeds 4 m/s, and it returns to LQR only when the gust falls below 3 m/s. This 1 m/s hysteresis band prevents rapid back-and-forth toggling in the presence of small variations in gust magnitude. Because the gust input is deterministic in the present simulations and the switching is triggered only once per gust event, no chattering or oscillatory switching behavior is observed in any scenario.

### 5.1. LQR

For the generic state space model presented in equation 21, the controller is described as

(22) $$u=-k\cdot \overset{⃑}{x}\left(t\right)$$

The feedback gain *k* appearing in the preceding equation is obtained through the linear quadratic regulator design framework. The associated quadratic performance index for the LQR formulation, as described in [33], is appended as

(23) $$J=\;\int_{0}^{\infty }\left(\;x^{t}Qx+u^{T}Ru\right)\;dt$$

where “Q = Q ≥ 0 and R = R > 0 are the weighing matrices. The solution to the LQR problem, in the form of a gain matrix *k*, that minimizes cost, is achieved by solving the discrete-time Riccati equation [33]. The gain matrix that minimizes the quadratic cost function is

(24) $$K=\left(\;\rho R+B^{T}PB^{-1}B^{T}PA\right)$$

We use the algebraic Riccati equation to find *P* as

(25) $$P=A^{T}PA+Q-A^{T}PB\;(\rho R+B^{T}P{B)}^{-1}B^{T}PA$$

In LQR, the closed-form solution via the Riccati equation eliminates the need for online optimization, making it computationally efficient [34]. Although the LQR controller offers better control effort efficiency compared to the H_2_ controller, it lacks robustness under adverse weather conditions, particularly during high-intensity gusts. Therefore, within the proposed control framework, LQR is employed exclusively under nominal conditions where gust speeds are ≤3 m/s.

### 5.2. H_2_ Controller

The H_2_ control framework is an optimal design methodology that improves system performance by minimizing the overall energy of the closed-loop response to disturbances and noise [35]. By minimizing the H_2_ norm of the closed-loop transfer function, the controller effectively reduces the energy of the system response to gust disturbances, thereby enhancing stability and efficiency in turbulent airflows. We choose H_2_ control in this study because it is particularly suitable for gust mitigation as it directly minimizes the influence of energy-like gust inputs on the UAV dynamics, ensuring efficient attenuation of gust-induced oscillations without excessive control effort.

**Figure 8:**
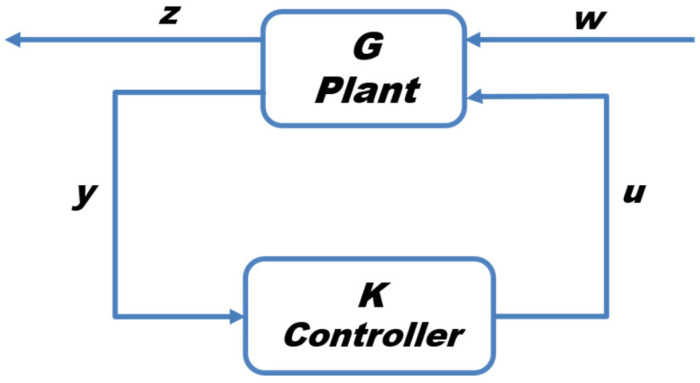
H_2_ control block diagram.

The closed-loop H_2_ configuration is shown in Figure 8, where *G* denotes the plant, *K* denotes the controller, *y* is the measured signal, *w* represents the disturbance, *u* is the control input, and *z* comprises all regulated terms. The H_2_ controller is designed to minimize the H_2_ norm [35]. The design process begins by formulating the transfer function, expressed as follows:

(26) $$G\left(s\right)\;=\;\left[\begin{array}{ccc} A & B_{w} & B_{u}\\ C_{y} & 0 & D_{yu}\\ C_{m} & D_{mw} & o \end{array}\right]$$

Several prerequisite conditions given below must be satisfied prior to the formulation of the H_2_ controller [36]:

• (*A,B_u_*) is stabilizable and (*C_m_,A*) is detectable.
• ${D_{yu}}^{*}\times \left[\begin{array}{cc} C_{y} & D_{yu} \end{array}\right]=\left[\begin{array}{cc} 0 & I \end{array}\right]$.
• $\left[\begin{array}{c} B_{w}\\ D_{mw} \end{array}\right]\times {D_{mw}}^{*}=\left[\begin{array}{c} 0\\ I \end{array}\right]$.
• $\left[\begin{array}{cc} A-j\omega I & B_{u}\\ C_{y} & D_{yu} \end{array}\right]$ has full column rank for all ω.
• $\left[\begin{array}{cc} A-j\omega I & B_{w}\\ C_{m} & D_{mw} \end{array}\right]$ has full column rank for all ω.

Satisfying the preceding conditions ensures the correct synthesis of the H_2_ controller. The standard H_2_ solution involves two Hamiltonian matrices, as expressed in Equations (27) and (28) [36].

(27) $$H_{2}=\left[\begin{array}{cc} A-B_{u}{\;\left({D_{yu}}^{*}\times D_{yu}\right)}^{-1}{D_{yu}}^{*}C_{y} & -B_{u}{\;\left({D_{yu}}^{*}\times D_{yu}\right)}^{-1}{B_{u}}^{*}\\ -{C_{y}}^{*}\{I-D_{yu}{\left({D_{yu}}^{*}\times D_{yu}\right)}^{-1}{D_{yu}}^{*}\}C_{y} & {-(A-B_{u}{\;\left({D_{yu}}^{*}\times D_{yu}\right)}^{-1}{D_{yu}}^{*}C_{y})}^{*} \end{array}\right]$$

(28) $$J_{2}=\left[\begin{array}{cc} (A-B_{w}{D_{mw}}^{*}{\;\left({D_{mw}\times D_{mw}}^{*}\;\right)}^{-1}{C_{m})}^{*} & -{C_{m}}^{*}{\;\left({D_{mw}\times D_{mw}}^{*}\;\right)}^{-1}C_{m}\\ -B_{w}\{I-{D_{mw}}^{*}{\;\left({D_{mw}\times D_{mw}}^{*}\;\right)}^{-1}D_{mw}\}{\;B_{w}}^{*} & A-B_{w}{{D_{mw}}^{*}\;\left({D_{mw}\times D_{mw}}^{*}\right)}^{-1}C_{m} \end{array}\right]$$

After determining the two Hamiltonian matrices, the corresponding variables X_2_ and Y_2_ are subsequently obtained using Equations (29) and (30).

(29) $${X_{2}=Ric\;(H}_{2})$$

(30) $${Y_{2}=Ric\;(J}_{2})$$

Finally, the H_2_ controller gain is calculated as follows [37]:

(31) $${\;K_{2}=\;-\left({D_{yu}}^{*}\;\times \;D_{yu}\right)}^{-1}\times \;{(B_{u}}^{*}\;X_{2}+\;{D_{yu}}^{*}C_{y})\;$$

### 5.3. Controller Switching Logic

To ensure a smooth transition between control laws, a gain-scheduling approach is adopted based on the magnitude of the gust disturbance. The controller switching architecture is illustrated in Figure 7. The switching mechanism operates within an intermediate disturbance regime, corresponding to gust velocities in the range of 3–4 m/s. The underlying concept is to continuously blend the LQR and H_2_ feedback gains through a scalar weighting function $w\left(v_{gust}\right)$, resulting in a combined switching gain $K_{sw}$ defined as follows:

(32) $$K_{sw}=\;\left(1\;-\;w\left(v_{gust}\right)\right)K_{LQR}+\;w\left(v_{gust}\right)K_{H_{2}}$$

(33) $$\begin{array}{cc} & 0\;\;\;\;\;\;\;\;\;if\;v_{gust}\;\leq \;v_{low}\;\;\left(\;LQR\right)\;\\ w\left(v_{gust}\right)\;\;\;\;\;=\; & \frac{v_{gust}-v_{low}}{v_{high}-v_{low}}\;\;\;\;\;if\;v_{low}<v_{gust}<v_{high}\;\;(Switching)\\ & 1\;\;\;\;\;\;\;\;if\;v_{gust}\;\geq \;v_{high}\;\;\left(\;H_{2}\right) \end{array}$$

where $v_{low}$ is 3 m/s and $v_{high}$ is 4 m/s.

## 6. Results and Discussions

The NFCD-installed ornithopter’s longitudinal model and the developed hybrid controller presented in the previous section are simulated and analyzed at different gust intensities in this section. The three control regimes respond exactly in line with the control architecture in Figure 7 at different simulated gust intensities. The optimal LQR controller is engaged under mild gust conditions (0.75 m/s, 1.5 m/s, 2.25 m/s and 3 m/s). The switching controller is activated for a moderate gust (3.5 m/s). For higher gust intensities (6 m/s, 9 m/s, 12 m/s and 15 m/s), the H_2_ controller operates alongside feather actuation in the proposed NFCD to mitigate high-intensity gusts. Further, the H_2_ synthesis assumptions listed in Section 5.2 are satisfied for the present system because the reduced-order bond-graph model yields a fully controllable and observable linear representation. Numerical rank tests confirmed that (A,Bu) is stabilizable and (Cm,A) is detectable, and the frequency-domain matrices involving A−jωI, Bu, Bw, Cy, and Cm retain full column rank across all relevant frequencies. These properties arise directly from the physically parameterized structure of the NFCD–ornithopter model and ensure the feasibility of LQR and H_2_ controller synthesis without requiring additional assumptions.

**Figure 9:**
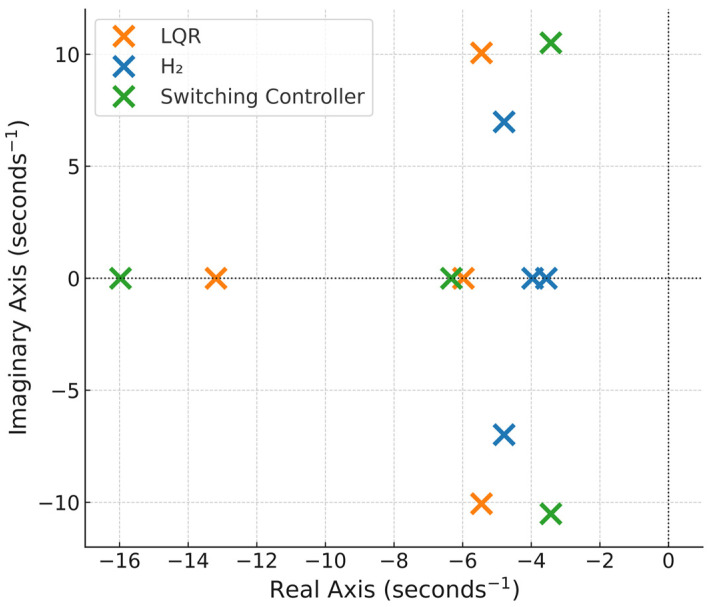
Closed-loop pole-zero plot of the hybrid LQR-H_2_ controller for different control regimes.

**Figure 10:**
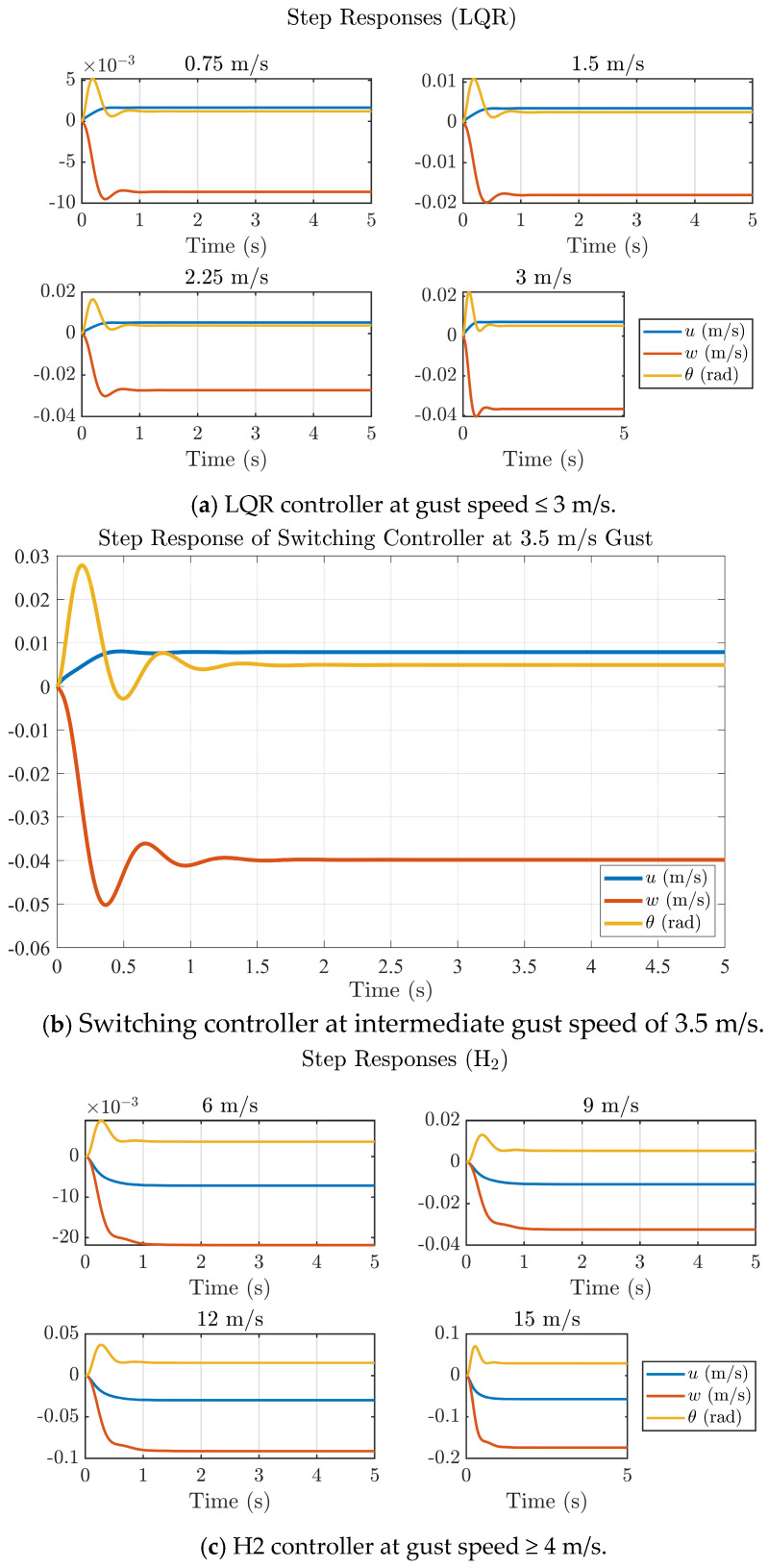
Controlled step response of hybrid LQR−H_2_ controller at different step gust speeds.

**Figure 11:**
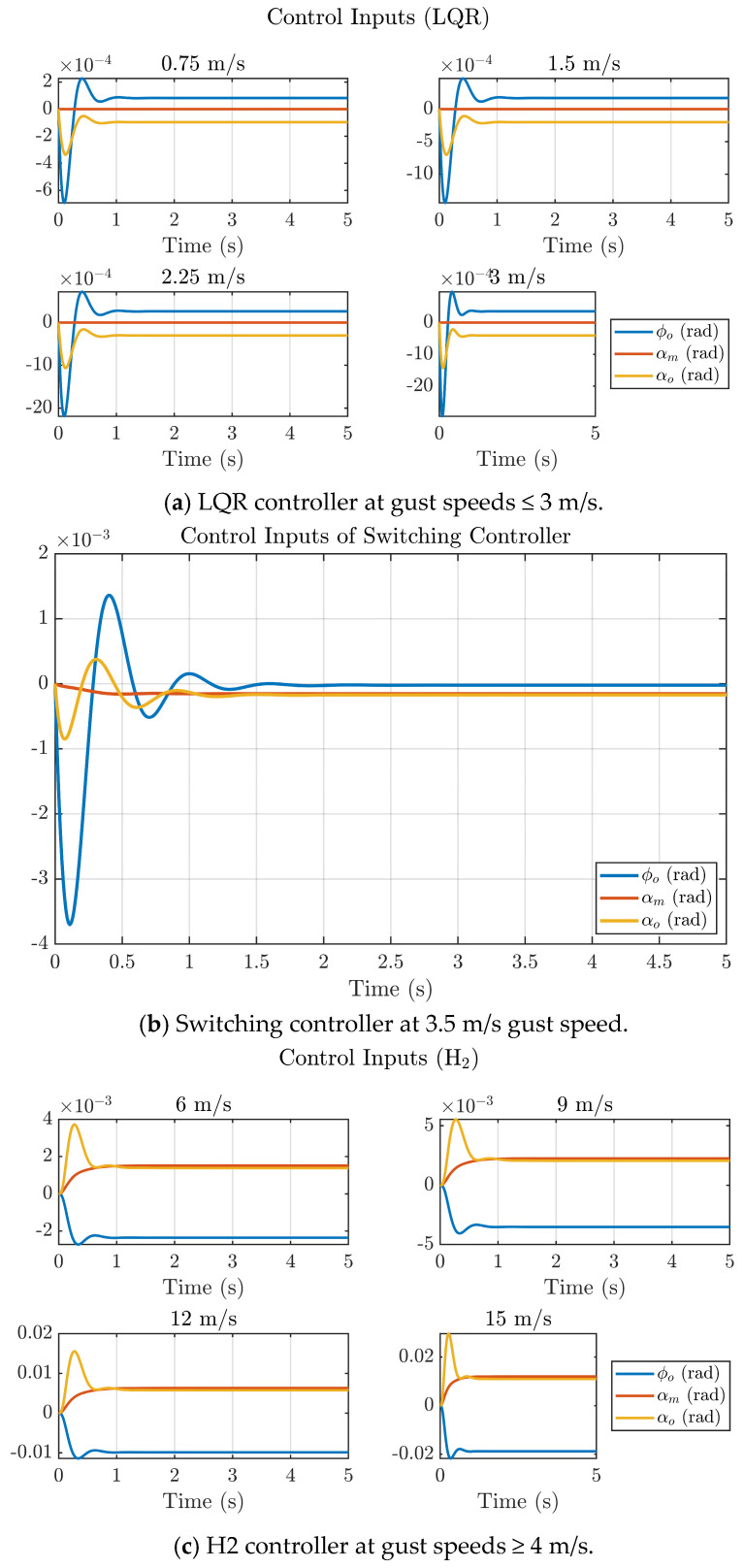
Control input plots of the hybrid LQR−H_2_ controller at different gust speeds.

**Figure 12:**
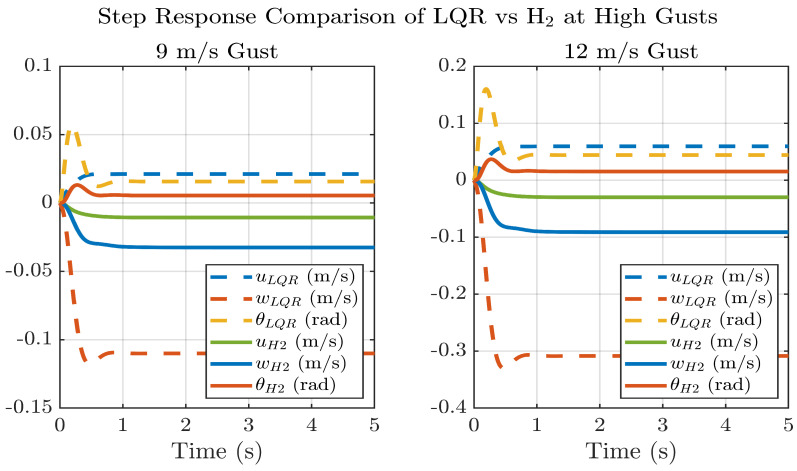
H_2_ vs. LQR at high gusts.

The NFCD modifies the local aerodynamic environment by allowing covert-feather deflection to relieve instantaneous pressure spikes during gust encounters. This passive-active interaction reduces the effective aerodynamic stiffness at the wing root and produces lower transient loads that the controller must reject. As a result, both LQR and H_2_ benefit from working on a dynamically ‘softer’ system: the LQR gain-set experiences reduced overshoot due to lower disturbance energy, while the H_2_ controller further attenuates the residual feather loads through its optimal disturbance-rejection structure. Therefore, the improved closed-loop performance demonstrated in Figure 9, Figure 10, Figure 11 and Figure 12 arises from the combined aerodynamic relief produced by the NFCD and the hybrid controller’s ability to stabilize the reduced post-gust loads.

The closed-loop pole-zero plots for the LQR, H_2_ and the switching controllers are shown in Figure 9, and the corresponding closed-loop eigenvalues are listed in Table 3. All eigenvalues lie in the left half plane (LHP), confirming that the unstable ornithopter system has successfully been stabilized by all three control regimes. The LQR design yields relatively faster dynamics with higher real parts, while the H_2_ controller shifts the poles closer to the imaginary axis, indicating smoother but slower transient behavior with enhanced damping. The switching controller exhibits a dominant pole at −15.97, representing a rapid stabilizing response when transitioning between low and high gust regions. The presence of complex conjugate poles in both LQR and H_2_ configurations signifies lightly damped oscillatory modes that are effectively attenuated under the hybrid control scheme.

Figure 10 illustrates the closed-loop step responses of the ornithopter model under LQR, H_2_, and switching control schemes for varying gust magnitudes. At low gust intensities (<3 m/s), the LQR controller maintains stable performance with small overshoots and rapid settling of the forward velocity (u), vertical velocity (w), and pitch angle (θ). However, it can be seen that as the gust intensity increases to 3 m/s, the LQR response exhibits slower damping and increased oscillations. In contrast, the H_2_ controller, evaluated over high gust intensities with gusts from 6 m/s to 15 m/s, provides smoother and more dampened responses with reduced peak deviations, indicating improved disturbance attenuation. The switching controller, activated in the intermediate gust regime (3–4 m/s), demonstrates a balanced dynamic behavior preserving the fast-transient characteristics of LQR while achieving the damping performance of the H_2_ design. Overall, the results confirm that the proposed hybrid LQR-H_2_ strategy enhances the ornithopter’s aerodynamic resilience, achieving stability with settling times less than 1.2 s across a broad range of gust disturbances.

Figure 11 illustrates the control input responses ($\varphi_{o},\;\alpha_{m},\;\alpha_{o})$ generated by the LQR, H_2_ and switching controllers under varying gust intensities. For low-gust conditions (≤3 m/s), the LQR controller produces small and smooth control deflections that settle within approximately 1 s, indicating efficient actuator usage with minimal transients. As gust magnitude increases (≥4 m/s), the H_2_ controller demands higher control effort, yet the signals remain well-damped and converge less than 1.2 s, confirming its enhanced disturbance-rejection capability without inducing oscillatory instability. The switching controller activated around 3.5 m/s exhibits the balanced performance, achieving rapid suppression of initial transients and settling in under 1.3 s while maintaining moderate control amplitudes. Overall, the hybrid LQR-H_2_ scheme ensures stable and energy-efficient electromechanical feather actuation of the NFCD, preserving smooth control transitions and avoiding actuator saturation even under intensified gust conditions.

Figure 12 compares the closed-loop step responses of the LQR and H_2_ controllers under high-intensity gusts of 9 m/s and 12 m/s. Both controllers achieve stable convergence within approximately 1.2 s, yet the H_2_ controller demonstrates noticeably faster damping and smaller steady-state deviations across all states. Quantitatively, the H_2_ controller reduces longitudinal velocity (*u*) deviations by ≈52% at 9 m/s and ≈51% at 12 m/s, vertical velocity (*w*) oscillations by ≈68% at 9 m/s and ≈71% at 12 m/s, and pitch-angle (*θ*) variations by ≈60% at 9 m/s and ≈67% at 12 m/s relative to the LQR case.

The results in Figure 10, Figure 11 and Figure 12 further enunciate that at nominal gusts (≤3 m/s), the LQR controller achieves the desired settling time (<1.2 s) with minimal NFCD actuation, thereby reducing actuator workload and sensor-noise amplification. This operational threshold follows the classification of low-intensity gusts adopted by [18], who applied LQR control effectively below 3 m/s gusts. In contrast, at higher gust levels (≥4 m/s), the H_2_ controller produces markedly smaller state excursions: approximately 50–70% reductions in *u*, *w*, and *θ* compared with LQR at 9–12 m/s, demonstrating superior disturbance attenuation. A scheduling transition within the 3–4 m/s range ensures smooth switching between controllers without command discontinuities. These findings further justify the hybrid control architecture and the design choice of distinct control regimes for different gust intensities, yielding energy-efficient performance of the ornithopter under nominal conditions and robust stability during strong gusts.

**Figure 13:**
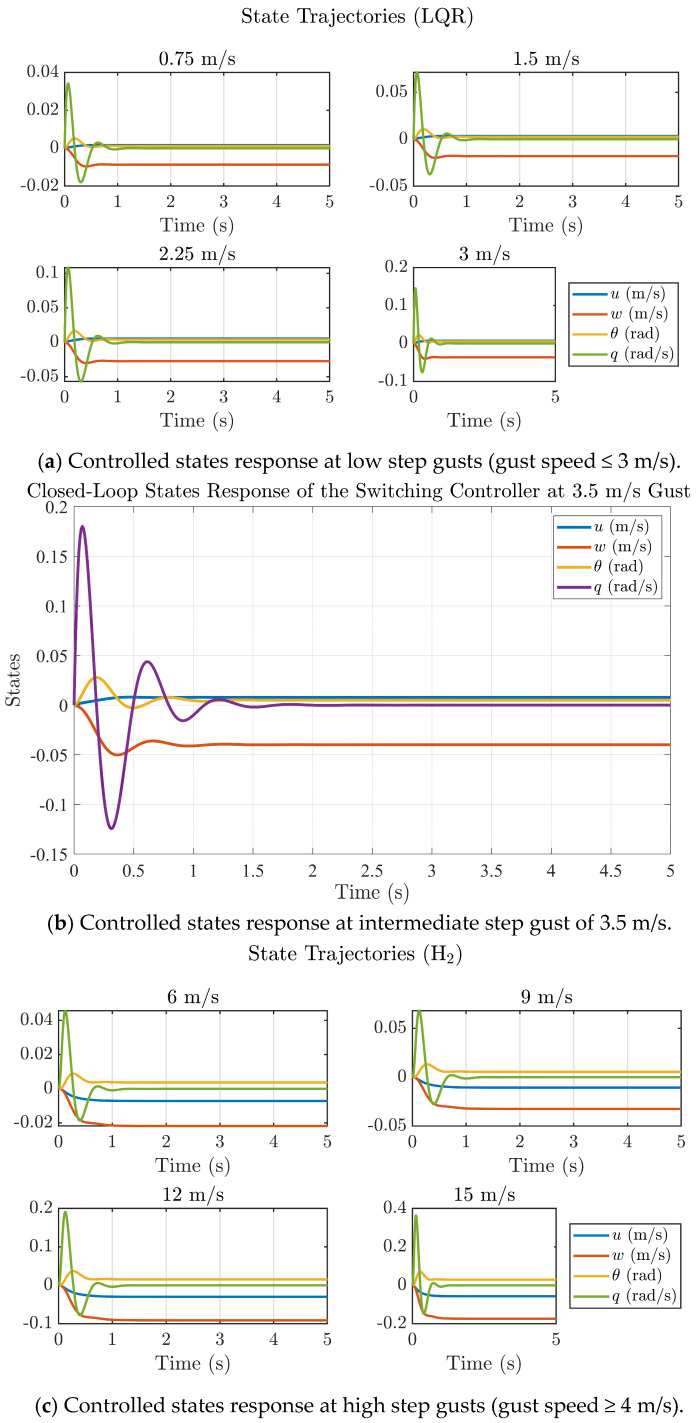
State response of the hybrid LQR−H_2_ controller at different step gust speeds.

Figure 13a–c present the dynamic state responses of the closed-loop ornithopter system equipped with the Nature-Inspired Flow Control Device (NFCD) under different step-gust disturbances. For low-gust conditions (≤3 m/s), the LQR controller ensures rapid convergence of all internal states (*u*, *w*, *θ*, *q*) with negligible overshoot, achieving complete stabilization within 0.95 s. In the intermediate gust regime (3.5 m/s), the switching controller provides a balanced response, combining the fast-transient performance of LQR with the damping characteristics of the H_2_ controller, settling in less than 1.4 s. Under higher gust intensities (6–15 m/s), the H_2_ controller effectively suppresses oscillations and maintains stable trajectories with smooth damping, settling in about 1.2 s despite increased disturbance levels. Overall, settling of states in all control regimes in less than 1.4 s matches the experimental results in the literature [38]. The results further demonstrate that all internal states remain bounded and converge quickly, confirming the hybrid controller’s ability to maintain flight stability and robustness across a wide range of gust conditions. The consistent settling trends also validate the accuracy of the reduced-order bond-graph model and the efficacy of the proposed hybrid LQR-H_2_ control framework for gust-mitigating ornithopter flight.

**Figure 14:**
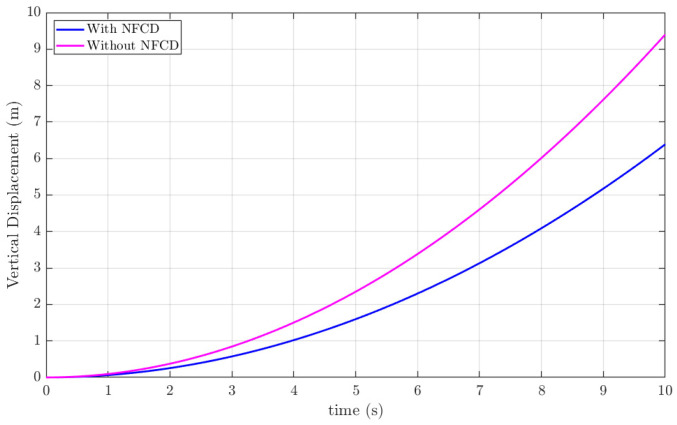
Ornithopter vertical displacement at 15 m/s gust.

Figure 14 compares the vertical displacement response of an ornithopter without an NFCD to that of an NFCD-equipped ornithopter controlled using a hybrid LQR-H_2_ scheme under a 15 m/s gust disturbance. The simulation results indicate that the combined action of covert-feather actuation and the hybrid controller achieves approximately 32% reduction in gust-induced effects. This 32% gust mitigation refers to the percentage reduction in the peak vertical aerodynamic force and the peak vertical displacement of the ornithopter when the NFCD is enabled, relative to the baseline case without NFCD, under the same gust magnitude. This metric quantifies the attenuation of the maximum gust-induced load rather than energy or RMS reduction. These results successfully certify the efficiency of the proposed NFCD augmented with a hybrid LQR-H_2_ controller in tackling gusts.

Table 4 presents a comparison between the 5 s simulation results of the present study and the experimental measurements reported in [39] at a gust speed of 15 m/s. In the experimental work, the aerodynamic force and resulting vertical displacement of an elastic wing were measured under controlled gust excitation in a Price–Païdoussis low-turbulence wind tunnel, following a combined XFLR5 aerodynamic and CATIA V5 structural aeroelastic modeling framework. A close agreement is observed between the present simulation and the experimental data, particularly in terms of force attenuation and vertical displacement. The significantly lower force and displacement in the “with NFCD” case further confirm the effectiveness of the proposed gust-mitigation mechanism. The consistency with experimentally validated behavior supports the accuracy and physical reliability of the proposed NFCD-installed ornithopter controlled with a hybrid LQR-H_2_ scheme in mitigating gusts.

Table 5 contrasts the proposed hybrid LQR–H_2_ framework with representative conventional, bio-inspired, and learning-based gust-alleviation methods reported in recent literature. Earlier approaches generally target mild disturbance environments or address only basic attitude stabilization, limiting their applicability under stronger gust loads. In comparison, the presented controller sustains a stable response in gusts up to 15 m/s and achieves roughly 70% attenuation of gust-induced oscillations, along with a 32% reduction in gust effects, while maintaining a settling time below 1.4 s. This level of disturbance rejection marks a clear performance elevation over existing ornithopter control strategies. The gains arise from the synergy between the LQR–H_2_ architecture and the NFCD’s bio-inspired flow-control behavior, which leverages fluid–structure interactions to dissipate incoming gust energy in real time, closely emulating natural avian mechanisms.

### Experimental Correlation and Validation

Although the present work is simulation-based and focuses on reduced-order modeling and control synthesis, overall, ornithopter behavior under 15 m/s gust shows strong correspondence with experimentally reported trends. Table 4 already provides a quantitative comparison with the gust tunnel experiments of Communier et al. [39], who measured aerodynamic forces and vertical displacement of an ornithopter under a 15 m/s gust using an elastic wing in the Price–Païdoussis low-turbulence wind tunnel. The present simulation exhibits force attenuation and displacement levels (with NFCD: 11.0 N, 1.56 m and without NFCD: 16.2 N, 2.3 m) very close to the experimental results (16.6 N, 2.4 m), validating the physical plausibility of the modelled gust response.

Additionally, the dynamic parameters for the electromechanical feather actuation loop, PZT stiffness, voice-coil force constant, and hinge damping are taken from previously reported bench-level tests, ensuring realistic actuator behavior. This strong correlation between simulated ornithopter and experimentally observed findings supports the validity of the reduced-order simulated model for the purpose of hybrid LQR–H_2_ controller evaluation. The parameters of the complete bond graph model are appended in Table 1 and are taken from the author’s previous work [25,26].

Although a full prototype fabrication is beyond the scope of this study, the integration of experimentally derived actuation parameters and validated aerodynamic response trends ensures that the hybrid LQR–H_2_ controller is evaluated on a physically meaningful baseline model. Future work will extend this validation through hardware-in-the-loop tests and controlled gust-tunnel experiments using a built NFCD-integrated ornithopter platform.

## 7. Conclusions

This study presents a hybrid LQR-H_2_ control framework for a kestrel-based ornithopter, incorporating an electromechanical Nature-Inspired Flow Control Device (NFCD) that emulates the adaptive deployment of covert feathers and is modeled using a reduced-order bond-graph approach. The hybrid control architecture employs an LQR controller for nominal gusts (≤3 m/s), an H_2_ controller for high-intensity gusts (≥4 m/s), and a gain-scheduled transition in the 3–4 m/s range. Simulation results show up to 70% reduction in gust-induced oscillations, 32% gust-mitigation efficiency, and stabilization within 1.4 s under severe gusts, confirming the robustness and responsiveness of the proposed system. The findings demonstrate that integrating biomimetic aerodynamics and nature-inspired flow control with advanced hybrid control enhances gust tolerance and overall flight stability in ornithopters.

Future research will extend the present reduced-order formulation by incorporating high-fidelity CFD-based aerodynamic characterization of the NFCD-equipped wing to capture unsteady effects such as LEV formation, added-mass forces, and wake–wing interaction. Multi-axis gust fields, flexible-wing aeroelastic coupling, and surrogate unsteady aerodynamic models will be integrated to construct a more comprehensive aerodynamic representation suitable for broader flight-envelope analysis. On the control side, parametric uncertainty modeling, observer-based state estimation, and data-driven/learning-based adaptive controllers are being explored to enhance robustness under broadband turbulence. In parallel, hardware-in-the-loop validation and controlled gust-tunnel testing are planned to experimentally demonstrate the hybrid LQR–H_2_ controller on a physical ornithopter prototype equipped with the NFCD. These steps will enable full verification of the proposed bio-inspired gust-mitigation strategy in realistic operating conditions.

## Figures and Tables

**Table 1:** Parameters of the bond graph model.

Components	Values
Motors	
Voltage source	7.2 V
Armature resistance of the motors	5.1 Ω
Gyrator ratio of motors	0.00813
Damping of motors	0.00068 N-s/m
Mass of motors	0.021 Kg
Gears	
Ratio of gears	0.112
Flapping Mechanism	
Mass moment of inertia of crank	0.009 Kg/m^2^
Transformer ratio of connecting rod	2
Transformer ratio of linkages	1
Mass of connecting rod	0.03 Kg
Mass moment of inertia of connecting rod	0.006 Kg/m^2^
Rigid Beam Wing	
Mass of rigid beam	0.4 Kg
Mass moment of inertia of rigid beam	0.024 Kg/m^2^
Transformer ratio of rigid beam	1
Main Body	
Mass of body	0.15 Kg
Mass moment of inertia (J_x_, J_y_, J_z_)	0.002, 0.004, 0.003 Kg/m^2^
Gust speed	25 m/s
Flap	
Mass of flap	0.018 kg
Mass of skeletal structure	0.098 kg
Gust velocity on feather	15 m/s
Voice Coil Actuator	
Inductance	0.89 H
Stiffness	0.589 KN/m
Piezoelectric Stack	
Resistance between amplifier and PZT	5 Ω
Mass of Stack	0.008 Kg
PZT spring stiffness	0.024 kN/m
PZT equivalent capacitance	1.5 × 10^−7^ F
Coupling Ratio	0.478
Spring	
Spring stiffness	0.03 kN/m
Mechanical Linkage	
Transformer Ratio	0.2

**Table 2:** Parameters of the LTI model.

Parameters	Description	Units
u	Body forward velocity	m/s
w	Body vertical velocity	m/s
θ	Body pitch angle	rad
q	Body pitch rate	rad/s
$\varphi_{o}$	Flapping stroke offset	rad
$\alpha_{m}$	Pitch angle magnitude	rad
$\alpha_{o}$	Pitch angle offset	rad

**Table 3:** Closed-loop eigenvalues.

LQR	H_2_	Switching Controller
−5.98	−3.56	−15.97
−13.19	−3.96	−6.33
−5.45 ± 10.06i	−4.79 ± 6.98i	−3.43 ± 10.51i

**Table 4:** Comparison of 5 s simulation response of present study with experimental results at 15 m/s gust speed.

		Force (N)	Vertical Displacement (m)
Present Work	With NFCD	11.0	1.56
	Without NFCD	16.2	2.3
Experimental [39]		16.6	2.4

**Table 5:** Comparison of present hybrid controller with literature.

Research	Method	Disturbance Type	Disturbance Rejection Results	Settling Time
Kim et al. [10]	RL and Strain sensors	3–7 m/s	Adaptive and stable flight	5 s
Yu et al. [15]	Reinforcement Learning and Hybrid RL–PD	Extreme attitudes (flips, aggressive maneuvers)	Stable recovery and reduced angular accelerations	1.5 s
Wang et al. [16]	Deep Reinforcement Learning	Level 5 winds (~8–10 m/s)	A 65% reduction in tracking error	2 s
Bhatia et al. [18]	LQR	Up to 3 m/s	Increased attitude stability and reduction in oscillations	1.8 s
Z heng et al. [38]	Model Predictive Control	Up to 0.25 m/s	Rapid convergence to reference	3 s
Proposed	Hybrid LQR–H_2_	15 m/s	Up to 32% gust mitigation	1.4 s

## Data Availability

The data that support the findings of this study are available from the corresponding author, upon reasonable request.
